# Characterisation of Calcium Phosphate Crystals on Calcified Human Aortic Vascular Smooth Muscle Cells and Potential Role of Magnesium

**DOI:** 10.1371/journal.pone.0115342

**Published:** 2015-01-21

**Authors:** Loïc Louvet, Dominique Bazin, Janine Büchel, Sonja Steppan, Jutta Passlick-Deetjen, Ziad A. Massy

**Affiliations:** 1 INSERM U-1088, Amiens, France; 2 University of Picardie Jules Verne, Amiens, France; 3 Université Pierre et Marie Curie, Collège de France, Paris, France; 4 Fresenius Medical Care Deutschland GmbH, Bad Homburg, Germany; 5 Department of Nephrology, University of Dusseldorf, Dusseldorf, Germany; 6 Paris Ile de France Ouest (UVSQ) University, Paris, France; Brigham and Women’s Hospital, Harvard Medical School, UNITED STATES

## Abstract

**Background:**

Cardiovascular disease including vascular calcification (VC) remains the leading cause of death in patients suffering from chronic kidney disease (CKD). The process of VC seems likely to be a tightly regulated process where vascular smooth muscle cells are playing a key role rather than just a mere passive precipitation of calcium phosphate. Characterisation of the chemical and crystalline structure of VC was mainly led in patients or animal models with CKD. Likewise, Mg^2+^ was found to be protective in living cells although a potential role for Mg^2+^ could not be excluded on crystal formation and precipitation. In this study, the crystal formation and the role of Mg^2+^ were investigated in an *in vitro* model of primary human aortic vascular smooth muscle cells (HAVSMC) with physical techniques.

**Methodology/Principal Findings:**

In HAVSMC incubated with increased Ca x Pi medium, only calcium phosphate apatite crystals (CPA) were detected by Micro-Fourier Transform InfraRed spectroscopy (µFTIR) and Field Effect Scanning Electron Microscope (FE — SEM) and Energy Dispersive X-ray spectrometry (EDX) at the cell layer level. Supplementation with Mg^2+^ did not alter the crystal composition or structure. The crystal deposition was preferentially positioned near or directly on cells as pictured by FE — SEM observations and EDX measurements. Large µFTIR maps revealed spots of CPA crystals that were associated to the cellular layout. This qualitative analysis suggests a potential beneficial effect of Mg^2+^ at 5 mM in noticeably reducing the number and intensities of CPA µFTIR spots.

**Conclusions/Significance:**

For the first time in a model of HAVSMC, induced calcification led to the formation of the sole CPA crystals. Our data seems to exclude a physicochemical role of Mg^2+^ in altering the CPA crystal growth, composition or structure. Furthermore, Mg^2+^ beneficial role in attenuating VC should be linked to an active cellular role.

## Introduction

Patients suffering from atherosclerosis, diabetes and chronic kidney disease (CKD) often present with vascular calcification (VC) [1–3]. In adults with CKD including dialysis patients, cardiovascular disease including VC remains to be the leading cause of death. VC manifests itself as deposits of calcium phosphate complexes accompanied by phenotypical cell fate changes in both the intimal and medial layer of the large arteries including the aorta, the myocardium as well as cardiac valves. VC is clinically reflected by changes in parameters such as pulse pressure, coronary artery calcification, intima media thickness or pulse wave velocity [4, 5]. The process of VC is not fully understood, but likely to be multifactorial [6]. It appears to be a tightly regulated, active, cellular process rather than just a mere passive precipitation of calcium phosphate, that somewhat resembles normal bone formation. How the process of calcification is initiated has been the subject of several studies. Some results suggest that matrix calcification could be initiated through passive calcium phosphate deposition and that it could be the formation of calcium phosphate nanocrystals that triggers the osteogenic changes that are associated with the accumulation of extracellular, crystalline structures [7, 8], whereas other studies show that calcification could be initiated by the release of matrix vesicles and/or apoptotic bodies from (dying) VSMC that act as nucleation points for forming crystals [9].

Some studies focused on characterising the anatomical, chemical and crystalline structure of the calcium phosphate deposits in patients or animal models with CKD, but the results remain heterogeneous. A study in uraemic patients revealed hydroxyapatite in non-visceral and arterial calcification, whereas visceral calcification was identified as an amorphous or microcrystalline compound composed of calcium, magnesium and phosphorus [10]. Another study assessed mineral deposits in the aortic wall in calcitriol- and non-calcitriol-treated rodent models of uraemia-induced VC. Here, they were composed of amorphous calcium phosphate precipitate, apatite and also whitlockite in animals treated with calcitriol [11]. In contrast, an other investigation reported a colocalization of hydroxyapatite and whitlockite in three out of six tissue samples taken from iliac arteries of uraemic patients [12], whereas another recent study on tissue specimens from non–CKD and CKD stage 2–4 patients undergoing carotid endarterectomy for severe atherosclerosis showed that in microcalcifications most of the calcium phosphate was amorphous rather than mature apatite. Moreover, it revealed that the relative contributions of whitlockite and calcium phosphate to microcalcification differed between patients with normal and mildly impaired renal function: Whereas in the samples of patients with normal renal function, whitlockite and calcium phosphate contribute to the mineral phase to an almost identical extent, approximately two-thirds of the mineral consists of calcium phosphate in the patients with impaired renal function [13].

In the course of VC vascular smooth muscle cells (VSMC) that compose the medial layer of the vascular wall are playing a key role. Recently, the effect of magnesium on VSMC transformation and calcification was assessed in vitro as well in as in the aortas of rodents. Here, magnesium was able to inhibit the calcification process and attenuated osteogenic differentiation of cells as reflected through an increased expression of anti-calcification proteins such as matrix gla protein, osteopontin and bone morphogenetic protein 7 [14]. These findings were confirmed by Salem *et al.*, who described correlations between magnesium, inhibition of VC in calcification-induced aortic rings of rats and clinical biomarkers [15]. To date, three studies directly addressed the effect of magnesium on VC of VSMC in vitro. Here, magnesium significantly reduced induced calcification both in bovine as well as human primary VSMC, downregulated known pro-calcification and upregulated anti-calcification markers towards restoring more physiological expression levels seen in uncalcified controls [16, 17, 18]. Moreover, an active, cellular, inhibitory role was attributed to magnesium as it was only able to exert its protective effect on living cells [17]. These findings do not exclude a potential passive role for Mg^2+^ ions in the onset of calcification, because in the presence of Mg^2+^ cumulating evidence shows that magnesium can have an inhibitory effect on hydroxyapatite formation and precipitation. Earlier *in vitro* work shows that magnesium is able to stabilize amorphous calcium phosphate and inhibits the formation of hydroxyapatite as well as calcium pyrophosphate dehydrate and calcium-acidic phospholipid-phosphate complexes [19–23]. In the literature, biological calcium phosphate crystals are often referred as hydroxyapatite leading this term to designate many complex compounds. Indeed, calcium phosphate hydroxyapatite is considered as a model compound for biological mineralization although its ideal formula has not actually been found *in vivo*. The composition of calcium phosphate apatite crystals has been shown to vary over a wide range due to the possibilities of anionic and cationic substitutions and the existence of different type of ion vacancies [24].

To our knowledge, for the first time, the crystal formation and the possible role of Mg^2+^ in VC through an *in vitro* model of primary human aortic vascular smooth muscle cells (HAVSMC) was investigated in this study. At this point, we have to underline that usual methods to gather information on the chemical nature of VC are based on staining procedures which display several limitations for identifying and quantifying apatite [25, 26]. All these limitations call for the need to combine usual techniques with physical techniques such as Scanning Electron Microscopy (SEM), Micro-Computerized Tomography, Micro-Fourier Transform InfraRed (µFTIR), RAMAN spectroscopies and X-ray fluorescence or X-ray scattering [27]. In this report, we developed an experimental procedure to culture cells on a support that was suitable to perform these techniques. The use of HAVSMC allows a direct assessment of the crystal formation process in a living system albeit under controlled conditions. In a novel way, the ultrastructure as well as the morphology of the calcium phosphate deposits directly on the culture cell layer were characterised with physical techniques such as Field Effect Scanning Electron Microscopy (FE—SEM), Energy Dispersive X-ray spectrometry (EDX) and µFTIR spectroscopy.

## Material and Methods

All chemicals were purchased from Sigma unless otherwise stated.

### Cell culture of HAVSMC

Primary human VASMC were isolated in our laboratory from explants of human aortic tissue obtained with appropriate ethical approval. The samples were obtained after aortic valve bypass surgery or other types of surgery on the aorta from patients with various cardiovascular diseases (Pr Caus, Pôle Coeur Thorax Vaisseaux, CHU Amiens, France). This protocol was approved by the Ethics Committee that is “Comité de Protection des Personnes (CPP) Nord-Ouest II” under ID #2009/19 to collect aortas and is valid for any experimental procedure. Briefly, as approved by the CPP (i.e. French Ethical Committee), the donors receive before surgery an oral and a written information explaining that if they don’t give their refusal, their aorta will be used for experimental purposes. Indeed, in regard to French law, the aorta is once removed considered as biological waste. Consequently, a written consent from the donor is not required. The investigations were performed according to the principles outlined in the Declaration of Helsinki for use of human tissue or subjects. The aortas were anonymized once removed from the donors in the surgical block and the samples were labelled with a number later in the laboratory for experimental convenience. The donors could not then be identified. As previously described [17], medial tissue was separated from segment of human aorta after removal of endothelium. Small pieces of tissue (1–2 mm^2^) were placed in culture dish in Dulbecco’s Modified Eagle’s Medium (DMEM) supplemented with 15% of fetal bovine serum (FBS, Dominique Dutscher), 4.5 g of glucose, 1 mmol/L of pyruvate, 100 U/mL of penicillin, and 100 µg /mL of streptomycin in a 5% CO2 incubator at 37°C. Cells that migrated from explants were collected when confluent. The cells were maintained in DMEM supplemented with 15% FBS, the medium was replaced twice a week. HAVSMC were identified by their typical hill and valley morphology, and purity of the primary cell culture was further checked by immunocytochemistry using a monoclonal antibody against the α-smooth muscle actin protein 1A4 (Acta 2) (Santa Cruz Biotechnology). α-smooth muscle actin 1A4 is an isoform typical of smooth muscle cells (SMC) and is present in high amounts in vascular SMC [28]. The cells were used between passages 6 and 10, during which time they were able to calcify. HAVSMC were pre-tested for calcification on classical cell culture plates prior use in any experimental set. Cells isolated from 4 independent donors were tested alternatively depending on the availability of cells requested for the various experiments. For calcification assays, cells were seeded at 150 000 cells / well on precut MirrIR slides (Kevley Technologies) placed in 6-well plates. Cells were treated for the indicated times (i.e. 14 and 18 days) with various media conditions from 2–3 days after plating.

### Calcification assays and sample preparation on MirrIR slides

MirrIR slides were cut into 20×25-mm pieces, sterilised in ethanol and dried in a laminar flow hood before use with cellular culture. To improve cell attachment and survival of HAVSMC on MirrIR slides, the slides were coated with gelatine 0.2% overnight in the incubator. The slides were then placed in 6-well plates while removing excess of gelatin solution, before seeding the slides with the cell suspension. The cell viability and attachment were greatly improved without affecting spectroscopic assays [29] comparing to cell growth on uncoated slides.

Calcification assays were conducted in 1% FBS DMEM. The control condition was incubated with 1% FBS DMEM only. DMEM medium is initially containing 0.9 mM of Pi, 1.8 mM of Ca^2+^ and 0.8 mM of Mg^2+^. When indicated, the media Pi and Mg^2+^ concentrations were increased using NaH_2_PO_4_ and MgCl_2_ supplementation, respectively. To induce calcification, Pi concentration was initially increased to reach 3mM during 10 and 14 days as performed in a previous study [17]. The weakness of the signal to detect Ca / Pi deposits in this experimental set (data not shown) led us to increase the Ca x Pi product for further experiments and to extend the incubation time in calcifying conditions to 14, 18 and 21 days. The Ca x Pi product in calcifying conditions was enhanced from 5.4 (initially with 3 mM Pi) to reach 7.2, using two different approaches: the samples were treated with 4 mM Pi (labelled Pi4), or they were incubated with a mix of 3 mM Pi and 2.4 mM Ca^2+^ (labelled PiCa). All experiments are summarized in table 1. For both types of calcification induction, Mg^2+^ was assessed at various concentrations including 1.5, 2 and 5 mM of total Mg^2+^ (labelled Mg 1.5, 2 and 5 respectively). At the end of the experiment, media were removed and cells were fixed using a 3.7% formaldehyde solution at room temperature for 15 minutes. As detailed in [30], the use of formaldehyde does not interfere with µFTIR. After removal of formaldehyde, the samples were dried and suitable for the µFTIR analysis. For some samples, a Von Kossa was performed before the analysis to allow a better visualization of the calcified areas.

**Table 1:** Identification of the samples studied through µFTIR experiments.

**donor**	**samples**	**VK staining**	**coating**	**incubation time**	**presence of apatite**
**I**	Ct	yes	no	14 days	no
	Pi3	yes	no	14 days	no
**II**	Ct	yes	no	14 days	no
	Pi3	yes	no	14 days	no
	Pi3 Mg2	yes	no	14 days	no
	Ct	no	no	14 days	no
	Pi3	no	no	14 days	no
	Pi3 Mg2	no	no	14 days	no
	Ct	no	yes	14 days	no
	Pi3	no	yes	14 days	no
	Pi3 Mg2	no	yes	14 days	no
	Ct	yes	yes	14 days	no
	Pi3	yes	yes	14 days	no
	Pi3 Mg2	yes	yes	14 days	no
**III**	Ct	no	yes	14 days	no
	Pi4	no	yes	14 days	yes
	Pi4 Mg2	no	yes	14 days	yes
	Pi4 Mg5	no	yes	14 days	yes
	Ct	no	yes	18 days	no
	Pi4	no	yes	18 days	yes
	Pi4 Mg2	no	yes	18 days	yes
	Pi4 Mg5	no	yes	18 days	yes
	Ct	yes	yes	21 days	no
	Pi4	yes	yes	21 days	no
	Pi4 Mg1.5	yes	yes	21 days	no
	Pi4 Mg2	yes	yes	21 days	no
	PiCa	yes	yes	21 days	no
	PiCa Mg1.5	yes	yes	21 days	no
	PiCa Mg2	yes	yes	21 days	no
	Ct	no	yes	21 days	no
	Pi4	no	yes	21 days	yes
	Pi4 Mg1.5	no	yes	21 days	yes
	Pi 4 Mg2	no	yes	21 days	yes
	PiCa	no	yes	21 days	yes
	PiCa Mg1.5	no	yes	21 days	yes
	PiCa Mg2	no	yes	21 days	yes
**IV**	Ct	no	yes	14 days	no
	PiCa	no	yes	14 days	yes
	PiCa Mg2	no	yes	14 days	yes
	PiCa Mg5	no	yes	14 days	yes
	Ct	no	yes	18 days	no
	PiCa	no	yes	18 days	yes
	PiCa Mg2	no	yes	18 days	yes
	PiCa Mg5	no	yes	18 days	yes

Samples nomenclature

**Ct** = samples incubated in 1% FBS DMEM

**Pi3** = samples incubated in 1% FBS DMEM with Pi set to 3 mM

**Pi4** = samples incubated in 1% FBS DMEM with Pi set to 4 mM

**Pi3 Mg2** = incubated in 1% FBS DMEM with Pi set to 3 mM and Mg^2+^ set to 2 mM

**Pi4 Mg1.5** = incubated in 1% FBS DMEM with Pi set to 4 mM and Mg^2+^ set to 1.5 mM

**Pi4 Mg2** = incubated in 1% FBS DMEM with Pi set to 4 mM and Mg^2+^ set to 2 mM

**Pi4 Mg5** = incubated in 1% FBS DMEM with Pi set to 4 mM and Mg^2+^ set to 5 mM

**PiCa** = samples incubated in 1% FBS DMEM with Pi set to 3 mM and Ca^2+^ set to 2.4 mM

**PiCa Mg1.5** = samples incubated in 1% FBS DMEM with Pi set to 3 mM, Ca^2+^ set to 2.4 mM and Mg^2+^ set to 1.5 mM

**PiCa Mg2** = samples incubated in 1% FBS DMEM with Pi set to 3 mM, Ca^2+^ set to 2.4 mM and Mg^2+^ set to 2 mM

**PiCa Mg5** = samples incubated in 1% FBS DMEM with Pi set to 3 mM, Ca^2+^ set to 2.4 mM and Mg^2+^ set to 5 mM

It is of note that these experiments were conducted concomitantly with and without cells. The table is indicating the presence of apatite in experiments with cells. Data of passive Ca / Pi deposition on MirrIR slides are discussed in a proper section.

For the Von Kossa staining, the cells were rinsed and incubated with a 5% AgNO_3_ solution for 30 min. Cells were further rinsed with water, incubated for 5 min in a photographic revelation solution, washed with water, then incubated 5 min with a 5% sodium thiosulfate solution to remove unreacted silver, washed again in water and finally dried. To evaluate passive Ca / Pi deposition, precut MirrIR slides were incubated as it was for samples with cells, slides were finally dried without further fixation.

### Specific controls

Media from the various experimental conditions were assessed for correct calcium, phosphorus and magnesium levels using an ADVIA 1800 Siemens autoanalyzer (Siemens Healthcare Diagnostics). No significant change in pH was observed at the various Pi and Mg^2+^ concentrations. This excludes a potential role of medium acidification in the observed decrease of mineralization. We excluded the potential action of the chloride ions in the MgCl_2_ salt used for these experiments on calcification reduction in a previous study [17].

### Field Effect Scanning Electron Microscope (FE—SEM)

A Zeiss SUPRA55-VP SEM was used for observation of microstructure. This field-effect “gun” microscope (FE-SEM) operates at 0.5–30 kV. High-resolution observations were obtained by 2 secondary electron detectors: an in-lens SE detector and an Everhart-Thornley SE detector. To maintain the integrity of the samples, measurements were taken at low voltage (between 0.5 and 2 kV) without the usual deposits of carbon at the surface of the sample. Energy Dispersive X-ray (EDX) experiments was also performed. In order to perform Ca^2+^ cartography, the FE-SEM operates at 12. kV.

### Energy Dispersive X-ray spectrometry (EDX)

The EDX detector is equipped with an ultra-thin window allowing detection of light elements. EDX provided the elemental composition of the solid phases and helped to identify them (point analyses and elemental maps).

### FTIR spectroscopy

µFTIR measurements were carried out on IN10 experimental device and also performed on an IN10MX microscope (Thermo Scientific) to allow recording of large maps. All spectra were collected in ultrafast mode using a 50 μm × 50 μm aperture. The spectra were collected in the 4000–800 cm^-1^ mid-IR range at a resolution of 16 cm^-1^ with one spectrum per pixel. Data analysis of IR spectra and chemical images was performed using OMNIC software (Thermo Scientific). In order to estimate the overall extent of mineralized matrix over the samples as well as its chemical nature, a first set of maps was performed (2000 µm x 4500 µm), then large maps were collected to allow an extended overview of the different conditions (3700 μm x 7700 μm). Due to the extended acquisition times to picture the maps, several maps were divided for convenience in 2 or 4 areas depending on the initial map size. Different options were applied to build the map: maximum of the peak without background estimated with different limits, evaluation of the area between different limits without background estimated with different limits. The maps which are presented correspond to the area calculated between 1000 and 1100 cm^-1^ without backgrounds. Data analysis of IR spectra and chemical images was performed using OMNIC software (Thermo Scientific).

## Results

### Preliminary results

Control conditions, i.e. samples with DMEM 1% FBS only, were assessed and showed a lack of characteristic FTIR signals corresponding to any of the Ca / Pi precipitates (amorphous carbonated calcium phosphate, whitlockite, hydroxyapatite…). Concerning the samples stained with Von Kossa, it appeared that this staining was not suitable for FTIR microspectroscopy on cell layer calcifications of our HAVSMC. While Von Kossa staining indicates the possible presence of calcium phosphate precipitates, µFTIR spectra clearly demonstrated that such dark deposits are not correlated to the presence of mineralized matrix. Indeed, no specific signal of Ca / Pi precipitates were detected following the staining regardless of the condition or incubation time observed (data not shown). Such observation is in line with previously published results [25]. In another experimental set, incubation time under calcifying conditions was extended to 21 days. However, results are presented in the S1 Fig. (IIA and IIB) but the long incubation time adversely affected the cell viability just as the massive mineral deposits observed prevented a discrimination between the different conditions. In this case, the thickness and the extent of the calcifications were too high to perform a reliable analysis or mapping of the FTIR measurement.

### Ca^2+^ and Pi induce the formation of hydroxyapatite crystals on HAVSMC cell layer

**Figure 1:**
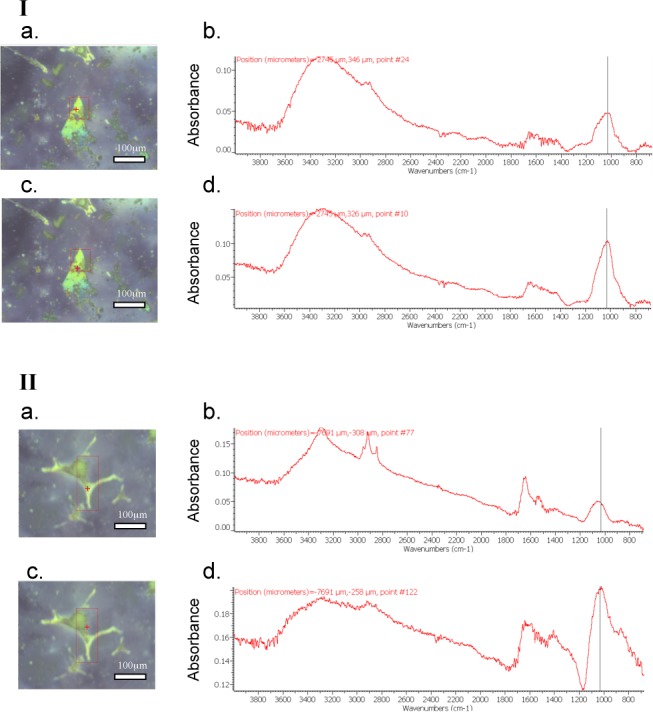
Typical optical image and mapping (scale from blue to red with increasing concentration), and FTIR spectra of crystals generated in Pi 4 or PiCa samples without or in presence of a total concentration of 2 mM of Mg^2+^. Ia) Optical image, and corresponding Ib) typical FTIR spectra, Ic) Optical image and corresponding Id) typical FTIR spectra of sample PiCa. IIa) Optical image and corresponding IIb) typical FTIR spectra, IIc) Optical image and corresponding IId) typical FTIR spectra of sample PiCa Mg2.

Regarding the nature of the mineral deposition, the characteristic absorption bands of the different calcium phosphate compounds are well assigned [31, 32]. A shoulder in the ν3 (P-O stretching vibration mode) absorption band explicitly indicates the presence of a carbonate calcium phosphate compound. Thus, major absorption bands are observed at 1037 cm^-1^ for apatite and 1068 cm^-1^ for amorphous carbonate calcium phosphate. In the case of whitlockite, two major absorption bands exist (1080 cm^-1^ and 1025 cm^-1^). As shown in Fig. 1, a single band corresponding to calcium phosphate apatite (CPA) (Ca_10−x+u□x−u_(PO4)_6−x_(CO3)_x_(OH)_2−x+2u_ with □ corresponding to vacancy, x≤2 and u≤x/2) crystals was detected in samples containing 3 mM Pi and 2.4 mM Ca^2+^ (sample PiCa) after 14 (data not shown) and 18 days (Fig. 1: Ib, Id) in calcifying condition. The same characteristic band was observed in samples containing 4 mM of Pi (S1 Fig.: Ia and Ic, respectively for 14 and 18 days in calcifying conditions). This means that the nature of the crystal was not influenced by the way we increased the Ca x Pi product to reach 7.2. By looking closely at the µFTIR profiles, a keen observer could notice that the peaks corresponding to CPA could not be perfectly superimposed. This fact is mainly due to the nature of the sample. More precisely, biological objects like mammalian cells could cause slight peak shifts or deviations compared to what is theoretically expected [33, 34, 35].

### Mg^2+^ does not modify Ca / Pi crystal structure and composition

Several publications show that the incorporation of foreign ions into hydroxyapatite structure can affect its crystallinity, morphology, lattice parameters, and stability [36, 37, 38]. Among them Mg^2+^ is known to inhibit the synthesis of hydroxyapatite and promote the formation of whitlockite in acellular systems [39, 40]. Therefore, HAVSMC were incubated under both calcifying conditions (Pi4 and PiCa) whilst Mg^2+^ was added to reach various final concentrations (1.5, 2 and 5 mM) during 14 (S1 Fig.: Ib) and 18 days. As shown in Fig. 1 (IIb, IId), the collected µFTIR spectra are pointing towards the presence of a single apatite-typical band regardless of the assessed Mg^2+^ concentration or calcifying condition (for Pi4 samples containing Mg^2+^; please see the S1 Fig.: Id). Absorption bands corresponding to whitlockite (Ca_9_Mg(HPO_4_)(PO4)_6_) were not detected in our samples meaning that the nature of the crystal was not affected by the presence of Mg^2+^. The µFTIR spectrum IIb in Fig. 1 tends towards the wavelength values of amorphous carbonate calcium phosphate. In addition to a slight peak shift that could be caused by the nature of the sample, this deviation could also reflect the presence of amorphous carbonate calcium phosphate mixed with CPA. Nevertheless, it would be an overstatement to impute the detection of amorphous carbonate calcium phosphate to the presence of magnesium because amorphous carbonate calcium phosphate is sporadically detected regardless of the presence of magnesium and does not represent the main crystal of interest here. Yet, a prior deposition of amorphous carbonate calcium phosphate is occurring during the CPA crystal formation process and throughout the crystal growth. As shown in the S1 Fig. (Ia, Ib, Ic, Id, IIAc, IIBc), the incubation time had no influence on the final crystal nature regardless of the condition. At 14, 18 or 21 days, the spectra are corresponding to CPA in Pi4 samples both in presence and absence of magnesium. It is of note that for 21 days samples (S1 Fig.: IIA and IIB) we restricted the map (S1 Fig.: IIAa, IIBa) to detect reliable µFTIR spectra. As mentioned in the preliminary results section, the calcifications were massive. These experimental conditions made it difficult to detect a reliable µFTIR peak and prevented a reliable µFTIR mapping. These results led us to consider the samples at earlier times of incubation in calcifying conditions to realise the µFTIR mapping.

### Location of calcium phosphate apatite on HAVSMC cell layer

**Figure 2:**
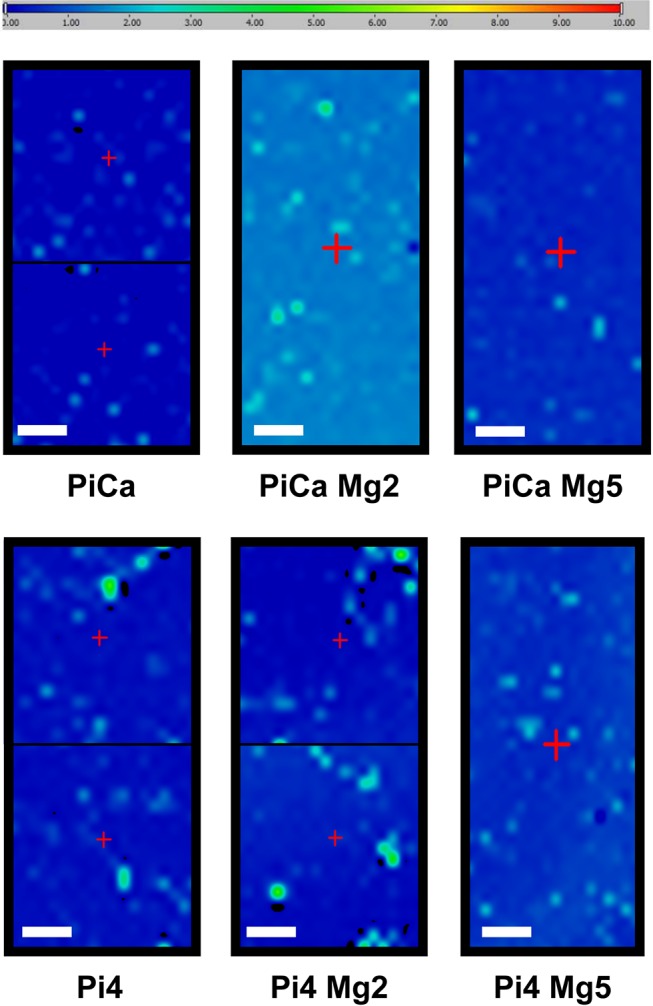
µFTIR maps obtained with calcium phosphate apatite features were collected for samples: PiCa, PiCa Mg2, PiCa Mg5, Pi4, Pi4 Mg2, Pi4 Mg5. The scale bar corresponds to 500 µm while the amplitude is between 0 and 10. Due to extended acquisition times, some maps were divided in 2 areas. The map size is 2000 µm x 4500 µm.

**Figure 3:**
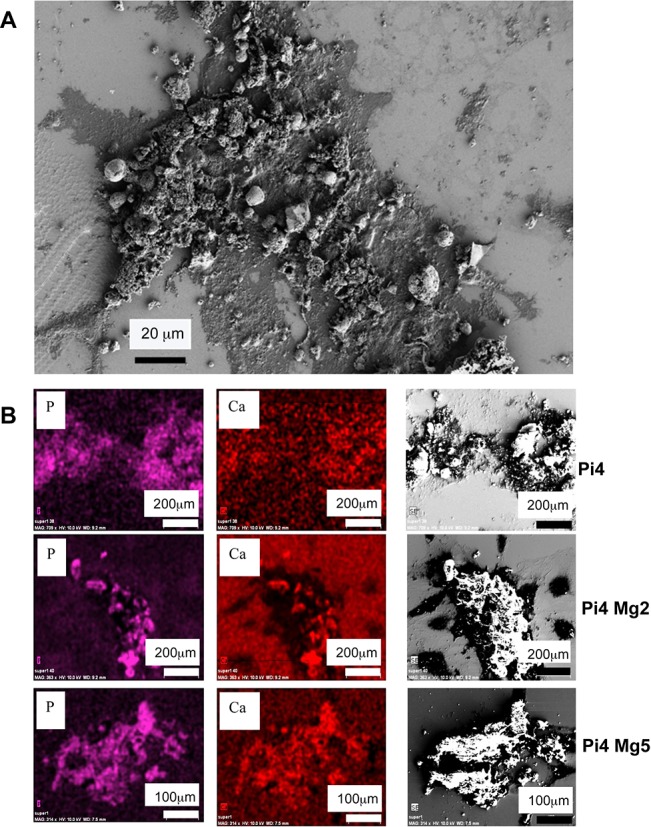
(A) SEM cartography of sample Pi4 Mg2 obtained with a magnification of 400 at 1 KeV (B) spatial distribution of Pi (purple maps), spatial distribution of Ca2+ (red maps) as obtained by EDX and SEM cartography (right pictures) obtained at 12 KeV for samples Pi4, Pi4 Mg2 and Pi4 Mg5.

To reveal the CPA distribution on the MirrIR slide surface, µFTIR maps were performed on samples incubated for 14 (data not shown) and 18 days under both calcifying conditions and Mg^2+^ supplementation as shown in Fig. 2. These maps are showing that the calcium phosphate deposits were not diffusely distributed but clearly appeared as spots located directly on the cells or in close vicinity thereof. No deposits were found in great spatial distance from any cellular feature. Similar spots were found regardless of the assessed calcifying condition and the supplemented Mg^2+^ concentrations (to reach 2 and 5 mM). This finding was further confirmed using Field Effect Scanning Electron Microscope (Fig. 3A) combined with Energy Dispersive X-ray spectrometry observations. In order to gather information regarding the spatial repartition of Ca^2+^ and Mg^2+^ cations as well as Pi anions, EDX experiments were performed. In Fig. 3B, purple (for Pi) and red (for Ca^2+^) maps perfectly matched, revealing a co-localization of Pi and Ca^2+^ in the same structure. Observations were similar regardless of the assessed Mg^2+^ concentration or the calcifying condition (data not shown for PiCa with or without supplemental Mg^2+^). Moreover, a supplemental deposit of Mg^2+^ apart from the one incorporated in the support was not detected by EDX avouching its absence from calcium phosphate deposits (data not shown). FE-SEM pictured clearly the presence of calcification preferentially positioned on cells (Fig. 3A and 3B) regardless of the assessed Mg^2+^ concentration or the calcifying condition (data not shown for PiCa with or without supplemental Mg^2+^). Characterizing the size and the shape of the CPA particles remained laborious because of their diversity. As shown in Fig. 3A, small round deposits were observed with sizes starting from approximately hundreds of nm to dozens of µm. Some of the more prominent deposits are pictured in Fig. 3B. They sized around hundreds of µm, their shapes were heterogenous and associated to the cellular layout.

### Passive Ca / Pi deposition on MirrIR slides

The passive Ca / Pi deposition was evaluated on MirrIR slides without cells. The slides were incubated as it was for samples with cells. Unlike to what was observed with living cells, the deposits were uniformly in nature and appeared far more prominently in conditions with increasing concentrations of Mg^2+^ (S2 Fig.).

### Effect of Mg^2+^ on crystal formation

**Figure 4:**
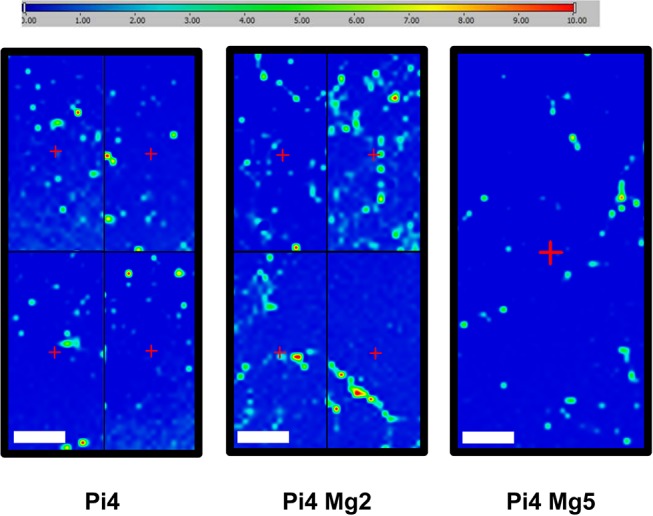
Large µFTIR maps obtained with Ca apatite features (between 1000cm-1 and 1100 cm-1) were collected for samples Pi4, Pi4 Mg2, Pi4 Mg5. The scale bar corresponds to 1000 µm while the amplitude is between 0 and 10. Due to extended acquisition times, some maps were divided in 4 areas. The map size is 3700 µm x 7700 µm.

As detailed above, µFTIR maps were performed to reveal where CPA deposition was preferentially occurring (Fig. 2). Looking closely at these maps, it appeared that raising Mg^2+^ up to 5 mM during 18 days seemed to reduce the number and intensity of CPA peaks compared to the calcifying conditions alone (Pi4 or PiCa). At day 14, such influence of Mg^2+^ was not noticeable (data not shown). Larger maps were then performed for the Pi4, Pi4 Mg2 and Pi4 Mg5 samples at day 18 to confirm this finding. In Fig. 4, the expanded µFTIR maps show a heterogeneous spatial repartition of CPA. Calcifications are present on all samples although the map corresponding to the condition containing 5 mM of Mg^2+^ is showing less numerous spots and is exhibiting CPA peaks of weaker intensities. It is of note that the µFTIR maps corresponding to the CPA signal represent a qualitative analysis of the calcium phosphate deposit and should not be interpreted as a quantitative analysis.

## Discussion

For the first time, the nature of the Ca / Pi deposits was described using physical techniques such as SEM, µFTIR, and EDX in *in vitro* induced mineralization by assessing directly the cell layer of primary HAVSMC. Previously, cell directed Ca / Pi deposition was inadequately studied and indirectly analysed in vascular cell culture models. For instance, Villa-Bellosta *et al.* recently performed X-ray diffraction, scanning electron microscopy, energy dispersive spectroscopy and transmission electron microscopy on ground powders from rat aorta smooth muscle cells [7], which only partly allow to draw conclusions about the cellular involvement in the initiation and development of the calcifications. Identically, Sage *et al.* assessed high Pi medium Ca / Pi nanocrocrystal pellets, but not crystals taken directly from the cell culture supernatant or the cell layer, with scanning electron microscopy, energy-dispersive spectroscopy and atomic force microscopy [8]. Nevertheless, Ca / Pi deposition was never studied directly within a cultured cell layer context. Moreover, the potential role of magnesium on the nature, formation, structure of nanocrystals has never been investigated with physical techniques at the cellular level.

The first major result was on the nature of the mineral deposition that was found in our HAVSMC incubated with increased Ca x Pi medium. Raising Pi alone or altogether with Ca^2+^ led to the same final calcium phosphate crystal structure: calcium phosphate apatite (CPA). CPA, which is the mostly found biological apatite is a non-stoichiometric apatite (Ca_10−x+u□x−u_(PO4)_6−x_(CO3)_x_(OH)_2−x+2u_ with □ corresponding to vacancy, x≤2 and u≤x/2): the presence of anionic (OH^−^) and cationic (Ca^2+^) vacancies has been evidenced compared to stoichiometric hydroxyapatite (Ca_5_(PO4)_3_(OH)) [27]. In biological systems, the term hydroxyapatite is given extensively to these kinds of apatites. CPA is the bone-like mineral that is commonly found in cultured osteoblastic models and is a marker of bone formation *in vitro* [25]. In the literature, VC was generally assumed to be composed mainly of hydroxyapatite [41]. More than a decade ago, several investigations demonstrated that coronary calcifications were already present in young dialysis patients, were progressing rapidly and were associated to a decreased survival [42, 43, 44]. Recently, the nature of arterial wall calcification from atherosclerotic and uremic patients was investigated more accurately with state-of-the-art physicochemical methods [12, 13, 45]. The results from these studies are showing that, apart from CPA, whitlockite (Ca_9_Mg(HPO_4_)(PO4)_6_) can also be found together with CPA in microcalcifications of arteries, but CPA is most prevalent in CKD patients. Likewise, similar results were found in arteries of CKD animal models [11]. In cell culture models, hyperphosphatemia or increased Ca x Pi lead to a transformation of vascular smooth muscle cells that allows mineral deposition. The studies from Sage *et al.* and Villa-Bellosta *et al.* are in line with our results as they reported the presence of CPA in respectively ground powders of mineral matrix and in high medium Pi centrifuged Ca / Pi nanocrystal pellets from rodent VSMC [7, 8]. In summary, our results are strengthening the hypothesis that a bone-like mineralized matrix is formed during the course of VC in the arterial wall.

In a previous study [17], we found that Mg^2+^ was able to partially prevent Pi-induced calcification in HAVSMC. This protective role was mediated by live cells through a modulation of various calcification markers. These findings did not exclude a potential passive role for Mg^2+^ in the onset of calcification, by either inhibiting nucleation, growth of calcium phosphates or altering crystal composition and structure. Several *in vitro* works studied the influence of Mg^2+^ on calcium phosphate crystal initiation and growth in acellular systems. As underlined by Salimi *et al.* [39], Mg^2+^ cations have a marked inhibitory effect on hydroxyapatite growth. As discussed by Hamad and Heughebaert [46], traces of Mg^2+^ have a significant effect on nucleation and growth of calcium phosphates. These ions delay the conversion of amorphous calcium phosphates to more stable apatite-like phases [47]. These results could not be validated in our cellular system, albeit passive deposition was evaluated on MirrIR slides without cells. With respect to the tested conditions, the deposits were more pronounced than their respective cellular counterpart and were far more prominent in conditions with increasing concentrations of Mg^2+^ (data not shown). This observation rules out the possibility of a direct physicochemical inhibition of Mg^2+^ on nucleation or growth of CPA in our model. Concerning the Mg^2+^ influence on the crystal structure and composition, several publications show that the incorporation of foreign ions into hydroxyapatite structure can affect its crystallinity, morphology, lattice parameters, and stability [36, 37, 38]. Among them, Mg^2+^ is known to inhibit the synthesis of hydroxyapatite and promote the formation of whitlockite in acellular systems [39, 40]. In the presence of calcifying HAVSMC, regardless of the tested conditions, only CPA was detected using µFTIR in our samples even when 5 mM of Mg^2+^ was added. The passive deposition on MirrIR slides without cells did not yield the formation of withlockite even in presence of Mg^2+^ (data not shown). Succinctly, Mg^2+^ was not able to modify the crystal structure in our experimental setup. Our data are strongly reinforcing recent findings from De Schutter *et al.* that assessed a rat model of uremia treated with a magnesium-based phosphate binder [48]. VC was analysed in aortas as well as in femoral and carotid arteries using synchrotron X-ray µ-fluorescence and X-ray µ-diffraction. Uremic animals developed severe VC that was reduced in the aortas of animals of the Mg^2+^-containing phosphate binder group. Yet, the synchrotron analysis of VC indicated that mineral precipitates exclusively consisted of calcium hydroxyapatite. None of the samples contained whitlockite or any other type of calcium phosphate deposits in uremic rats regardless of the treatment. These findings strongly support a previous report by Massy *et al.* that analyzed VC in atheromatous lesions of ApoE^-/-^ uremic mice with FTIR. In their animal model, VC was composed of calcite and hydroxyapatite [49]. Shortly, supplemental Mg^2+^ was not able to alter the crystal structure neither in cellular nor in animal models. Taken together, our results suggest that the Mg^2+^ beneficial effects on VC are bound to cellular activities rather than physicochemical modifications of the calcium phosphate crystal.

The spatial distribution of the CPA crystals was investigated using µFTIR maps as well as FE-SEM combined with EDX spectrometry observations. The complete set of data led us to conclude that calcification occurs mainly as CPA spots located close to or directly on the HAVSMC bodies. In our experimental setup, CPA deposition was massive and the various crystal sizes start at the nm scale to largely exceed the µm scale. In this report, we are dealing with microcrystals rather than nanocrystals. For the small deposits, the crystals looked round while they took the shape of the cells in the larger ones, suggesting that respectively matrix vesicles or apoptotic bodies may serve as calcification nidus [50]. The calcification process seems more advanced in our work than in the studies that characterized nanocrystals in rodent VSMC cultures [7, 8]. Moreover, our experiments are highlighting a potential beneficial effect of Mg^2+^. At 5 mM, Mg^2+^ was noticeably able to reduce the number and intensities of CPA µFTIR spots. However, this observation must be taken with caution since only a qualitative analysis was performed. Combined with the results from our previous study [17], it is reasonable to think that this decrease of CPA spots might be linked to the improvement of HAVSMC viability, the modulation of calcification markers or is likely to be bound to cellular activities attributable to Mg^2+^.

As noted above, we conducted preliminary experiments to optimize the detection of mineralized matrix by µFTIR. Indeed, Ca / Pi concentrations were raised and incubation time was extended compared to the initial experimental setup published in [17]. We hypothesize that these modifications were imposed by the nature of the MirrIR slide support which is physically different compared to the standard cell culture plastic used in the previous study. During these tests, Von Kossa staining was assessed to allow a better visualization of mineralized deposits to easily target them for µFTIR analysis. Nevertheless, analysis of the dark deposits failed to detect any of Ca / Pi precipitates of interest. As the principle of Von Kossa staining is a chemical reaction in which silver ions react with phosphate, the resulting product is altered in its chemical nature which could adversely prevent from its detection by µFTIR. Anyway, none of the Von Kossa processed samples displayed Ca / Pi deposits detectable by µFTIR. Our results are confirming Bonewald *et al.* observations that reported a discrepancy between Von Kossa stained darkened nodules and the detection of hydroxyapatite. Among various calcifying models, the darkened nodules produced in MC3T3-E1 cells were tested as bulk samples using a FTIR spectrometer and failed to display features of Ca / Pi minerals. Transmission electron microscopy confirmed this finding. Consequently, the Von Kossa staining was withdrawn from our study as it appeared not suitable to perform the various physical analyses carried out in the present work.

The main limitation of this study lies in the rather limited number of samples that were assessed. This limitation is mainly due to: firstly, the requested/allowed time for the various physical techniques that were performed; and secondly, to the restricted number of cells that were available for the various experimental conditions because the primary cell cultures could not be expanded sufficiently to repeat each experiments several times. This clearly forbade us to realize a quantitative and statistical analysis of CPA spots that were detected between the various conditions. Furthermore, an accurate biochemical assessment of Ca^2+^ could not be performed from our samples because of the nature of the MirrIR slides that already include Ca^2+^. For these reasons, only a qualitative analysis of the composition, structure and distribution of the Ca / Pi crystals is presented here. Nevertheless, we succeeded in developing an experimental procedure to perform µFTIR / EDX / FE—SEM directly at the cell layer level in cultured HAVSMC. Consequently, this report might contribute largely to develop direct assessment of cellular models with physical techniques which is actually not common when browsing the bibliography.

In summary, our data are excluding a physicochemical role of Mg^2+^ in inhibiting the crystal growth or in altering the calcium phosphate crystal composition or structure in an in vitro model of HAVSMC culture. Furthermore, the observed qualitative reduction of CPA spots should be linked to an active cellular role of Mg^2+^ in attenuating VC. Whether the in vitro data collected in the present study also have relevance in clinical setting is matter of additional works. The use of magnesium as a drug to lower serum calcium and phosphorus and its effect on outcomes in CKD patients was detailed in [51]. To our knowledge, magnesium-containing phosphate binders have not yet been investigated for quantitative VC reduction in a controlled, prospective clinical setting. Likewise, supplemental in vitro investigations are currently ongoing and will further elucidate the cellular mechanisms by which Mg^2+^ is able to prevent VC.

## Supporting Information

S1 Fig I. Typical mapping (scale from blue to red with increasing concentration), optical image and FTIR spectra (from left to right, respectively) of crystals generated in Pi4 calcifying condition with or without magnesium (Mg2) at day 14 and day 18 of incubation representing: Ia, sample Pi4 at day 14; Ib, sample Pi4 Mg2 at day 14; Ic, sample Pi4 at day 18; Id, sample Pi4 Mg2 at day 18. The scale bar corresponds to 500 µm. II. Typical mapping (scale from blue to red with increasing concentration), optical image and FTIR spectra of crystals generated in Pi 4 calcifying condition with or without magnesium (Mg2) at day 21 of incubation representing: IIA, a) Typical mapping (scale from blue to red with increasing concentration), b) Optical image, and c) typical FTIR spectra of sample Pi4; IIB, a) Typical mapping (scale from blue to red with increasing concentration), b) Optical image, and c) typical FTIR spectra of sample Pi4 Mg2.
(PDF)

S2 Fig µFTIR maps (scale from blue to red with increasing concentration) of crystals generated in Pi4 or PiCa calcifying condition with or without magnesium (Mg2 and Mg5) at day 18 of incubation. The map size is of 3000 µm x 4000 µm.
(PDF)
