# Supplementary figures and images for: Characterisation of Calcium Phosphate Crystals on Calcified Human Aortic Vascular Smooth Muscle Cells and Potential Role of Magnesium
Source: PLoS One. 2015 Jan 21;10(1):e0115342. doi: 10.1371/journal.pone.0115342 (PMC4301909; doi:10.1371/journal.pone.0115342)

# I

a.

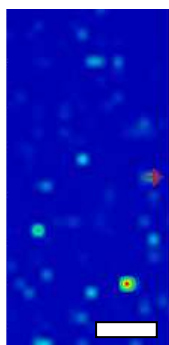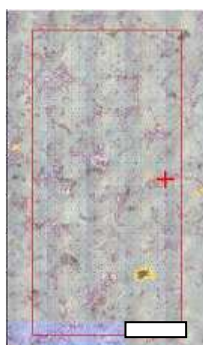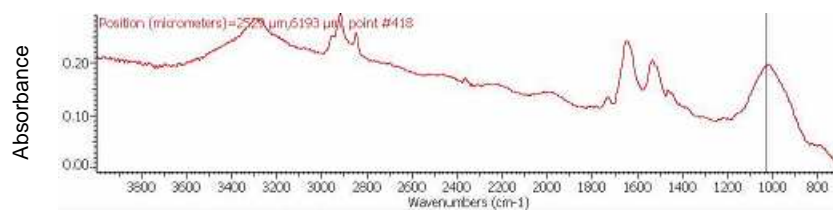

b.

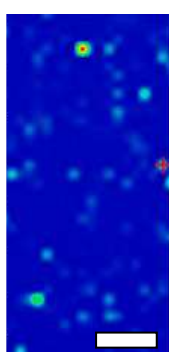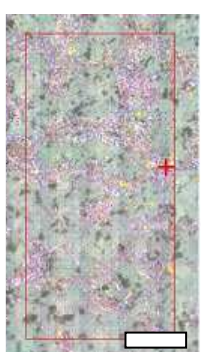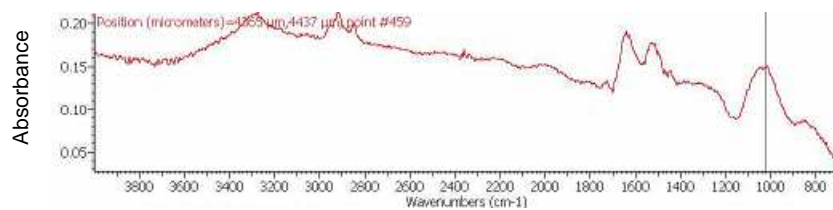

c.

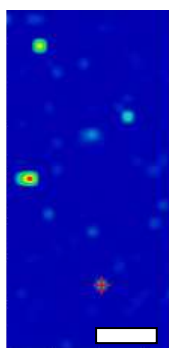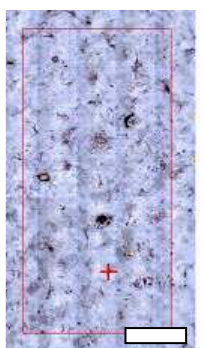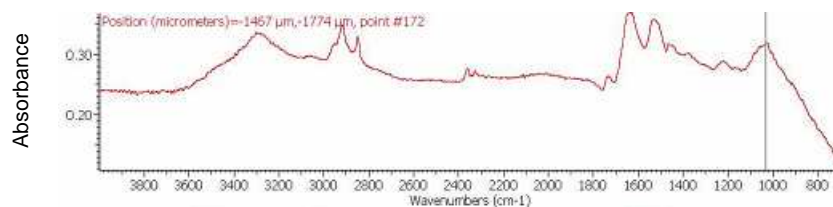

d.

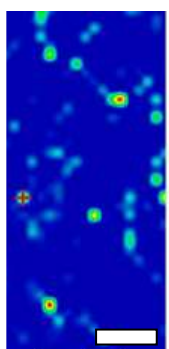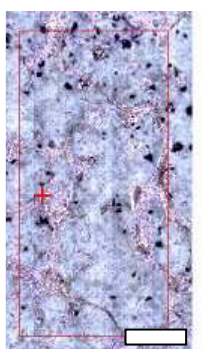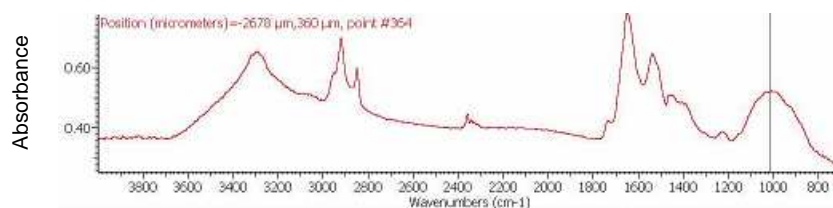

## II

### A

a

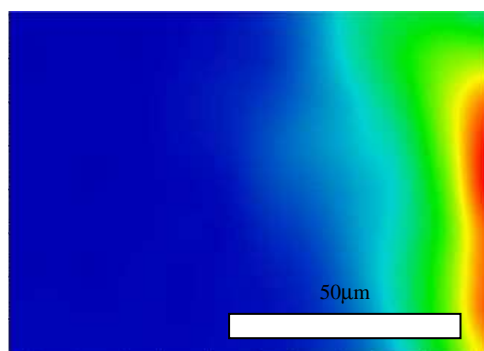

b.

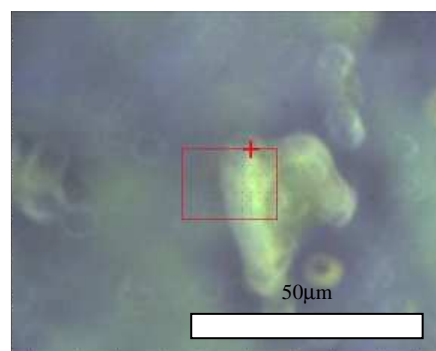

C.

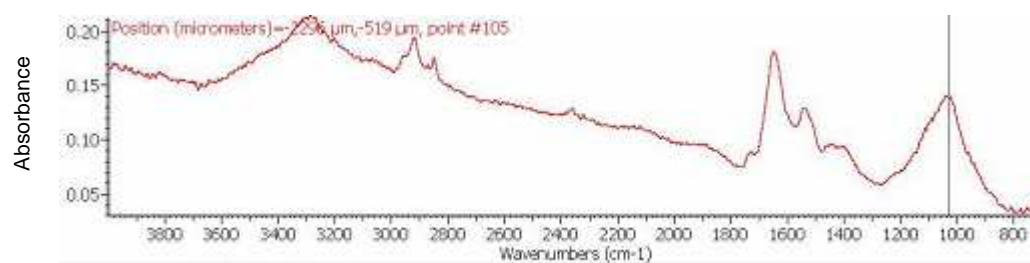

### B

a

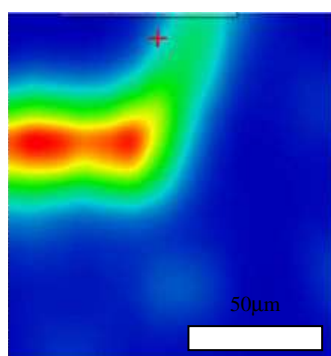

b.

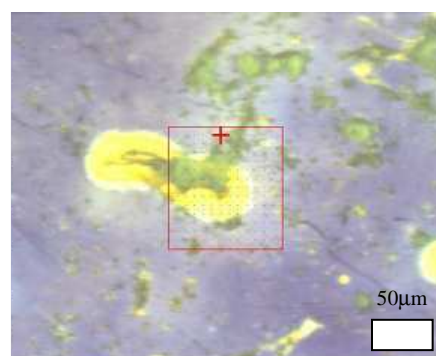

C.

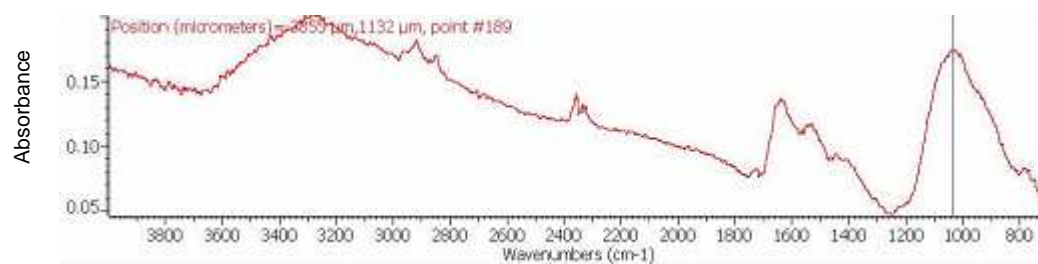

Supplement: S1 Fig — The scale bar corresponds to 500 µm. II. Typical mapping (scale from blue to red with increasing concentration), optical image and FTIR spectra of crystals generated in Pi 4 calcifying condition with or without magnesium (Mg2) at day 21 of incubation representing: IIA, a) Typical mapping (scale from blue to red with increasing concentration), b) Optical image, and c) typical FTIR spectra of sample Pi4; IIB, a) Typical mapping (scale from blue to red with increasing concentration), b) Optical image, and c) typical FTIR spectra of sample Pi4 Mg2. (PDF) [file pone.0115342.s001.pdf]

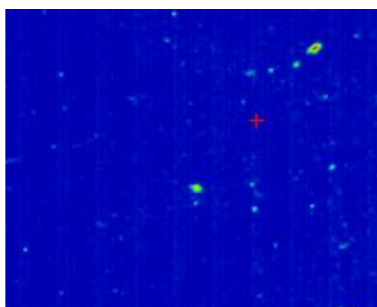

**Pi4**

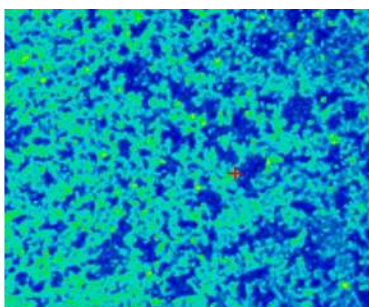

**Pi4 Mg2**

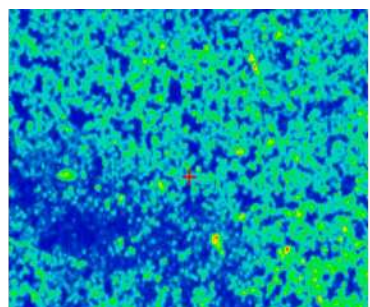

**Pi4 Mg5**

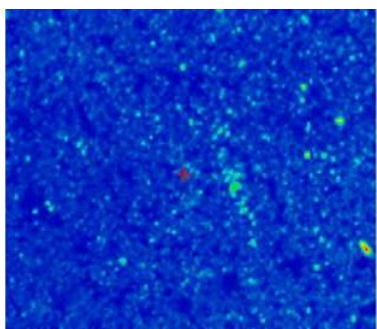

**PiCa**

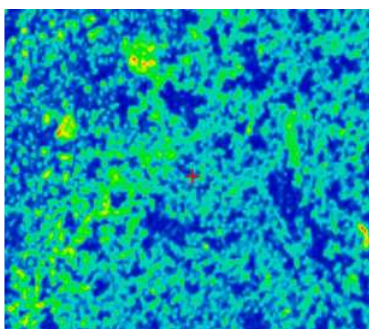

**PiCa Mg2**

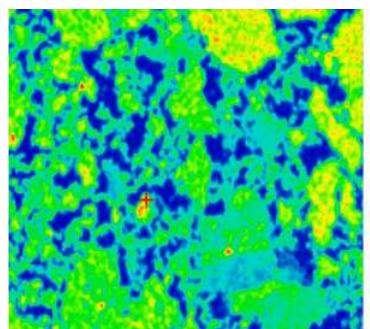

**PiCa Mg5**

Supplement: S2 Fig — The map size is of 3000 µm x 4000 µm. (PDF) [file pone.0115342.s002.pdf]
